# Expression Levels of Genes Encoding Proteins Involved in the Cell Wall–Plasma Membrane–Cytoskeleton Continuum Are Associated With the Maturation-Related Adventitious Rooting Competence of Pine Stem Cuttings

**DOI:** 10.3389/fpls.2021.783783

**Published:** 2022-01-20

**Authors:** Alberto Pizarro, Carmen Díaz-Sala

**Affiliations:** Departamento de Ciencias de la Vida, Universidad de Alcalá, Alcalá de Henares, Spain

**Keywords:** adventitious root meristem, cell division plane, cell polarity, conifers, vegetative propagation

## Abstract

Stem cutting recalcitrance to adventitious root formation is a major limitation for the clonal propagation or micropropagation of elite genotypes of many forest tree species, especially at the adult stage of development. The interaction between the cell wall–plasma membrane and cytoskeleton may be involved in the maturation-related decline of adventitious root formation. Here, pine homologs of several genes encoding proteins involved in the cell wall–plasma membrane–cytoskeleton continuum were identified, and the expression levels of 70 selected genes belonging to the aforementioned group and four genes encoding auxin carrier proteins were analyzed during adventitious root formation in rooting-competent and non-competent cuttings of *Pinus radiata*. Variations in the expression levels of specific genes encoding cell wall components and cytoskeleton-related proteins were detected in rooting-competent and non-competent cuttings in response to wounding and auxin treatments. However, the major correlation of gene expression with competence for adventitious root formation was detected in a family of genes encoding proteins involved in sensing the cell wall and membrane disturbances, such as specific receptor-like kinases (RLKs) belonging to the lectin-type RLKs, wall-associated kinases, *Catharanthus roseus* RLK1-like kinases and leucine-rich repeat RLKs, as well as downstream regulators of the small guanosine triphosphate (GTP)-binding protein family. The expression of these genes was more affected by organ and age than by auxin and time of induction.

## Introduction

Vegetative propagation has been employed mostly in commercial forests to propagate elite genotypes expressing yield traits of economic or ecological interest, such as wood quality or resistance to biotic or abiotic factors. Adventitious root formation by stem cuttings is an important step in successful vegetative propagation. The loss of competence to form adventitious roots impairs the propagation of high-quality genotypes for commercial production in breeding programs and forest plantations. In agronomy, the vegetative propagation of desired plant genotypes has been successfully used for centuries (Kovar and Kuchenbuch, 1994). However, in forestry, aside from a few genera, the vegetative propagation of elite trees has not been used extensively in most operational planting programs, even though many families need to be propagated using these procedures (Greenwood and Weir, 1995). Woody species are generally more recalcitrant to regeneration and propagation than herbaceous plants, and gymnosperms are more recalcitrant than many angiosperms (McCown, 2000; von Aderkas and Bonga, 2000). In trees, the decline in the capacity to form adventitious roots is a dramatic effect of age and maturation, and it affects the vegetative propagation of many tree species (Bonga et al., 2010). The basic nature of the maturation-related decline of adventitious root formation has been the subject of many investigations (Díaz-Sala et al., 1996; Goldfarb et al., 1998; Ballester et al., 1999; Greenwood et al., 2001; Day et al., 2002; Day and Greenwood, 2011; De Almeida et al., 2015). In conifers, this research has been performed using a simple experimental system that reproduce, in young seedlings and in short periods of time, the maturation-related decline of adventitious root formation in response to auxin that occurs in adult trees (Díaz-Sala, 2014, 2016). This system is based on the different rooting capacity of hypocotyl and epicotyl cuttings. In hypocotyl cuttings from very young seedlings of *Pinus taeda* and *Pinus radiata*, cambial and cambial-derived cells located centrifugally to the resin canals and closely associated with primary xylem poles, rapidly form a direct root meristem in response to exogenous auxin. The events leading to root formation include cellular reorganization, cell expansion and swelling during the initial 24–48 h of the root induction process, further asymmetric cell divisions, and the organization of a root primordium. The rooting capacity is lost in hypocotyl cuttings by the transition to secondary growth, in which the resin canals are included in the whole ring of the xylem. These poles are no longer evident after the formation of the epicotyl, an early phase of the maturation process in which the shoot makes the transition from an embryonic to a postembryonic pattern of development, and root formation in response to auxin is rare or lengthy (Díaz-Sala et al., 1996; Greenwood et al., 2001; Sánchez et al., 2007; Solé et al., 2008; Abarca et al., 2014). The initial response, i.e., the time course for cellular reorganization, cell expansion and cell division onset, and the rooting-cell mitotic frequencies, are similar in rooting-competent hypocotyl cuttings and non-competent hypocotyl or epicotyl cuttings (Díaz-Sala et al., 1996; Greenwood et al., 2001). However, the reorientation of the cell-division planes in the cambial-derived cells needed for the direct organization of a root meristem is barely induced in non-competent cuttings (Díaz-Sala et al., 1996; Goldfarb et al., 1998). Thus, the auxin priming action for rooting may be initiated before the initial proliferation event, even prior to the resumption of cell division, in rooting-competent cuttings. Three major cellular events are associated with the capacity to form adventitious roots in hypocotyl and epicotyl cuttings: (1) Modification of the division plane from the periclinal to anticlinal orientation (Díaz-Sala et al., 1996; Greenwood et al., 2001). The reorientation of cell-division planes during cell-plate formation is an evident change that characterizes rooting and rooting-derived cells in rooting-competent cuttings compared with the periclinal divisions of non-rooting cells or with the multiplicative periclinal divisions induced by auxin in non-competent cuttings; (2) Asymmetric auxin distributions and overlaps in the temporal and spatial distributions of auxin and *GRAS* genes before the resumption of cell divisions in the rooting cells (Sánchez et al., 2007; Solé et al., 2008; Abarca et al., 2014); and (3) Antagonism between adventitious rooting and cambium proliferation along with xylem formation (Ricci et al., 2016; Díaz-Sala, 2019; Pizarro and Díaz-Sala, 2020, Díaz-Sala, 2020).

The asymmetric auxin distribution in the rooting cells of competent hypocotyl cuttings is a major driver in adventitious root formation in pine (Abarca et al., 2014). Auxin maxima at the rooting cells during adventitious root formation may be driven by the dynamic redistribution of auxin carriers in rooting-competent cuttings. The polar distribution of PIN auxin carriers is maintained by cell wall–plasma membrane interactions (Feraru et al., 2011; Martinière et al., 2012), among others, which regulate the auxin distribution, and, as a consequence, root induction (Duman et al., 2020a,b). In addition, auxin redistribution may contribute to establish or sustain the mechanical or physical properties of cells that induce growth responses. The cell wall plays a central role in the control of growth and morphogenesis at cellular and tissue levels, integrating both mechanical and molecular regulatory processes (Wolf et al., 2012; Ali et al., 2014). Cell wall properties and modifications affect adventitious root formation in model, crop and tree species (Hutchison et al., 1999; Rigal et al., 2012; Lu et al., 2017; Li et al., 2018; Duman et al., 2020a,b; Eliyahu et al., 2020; Li, 2021). Although the emergence of lateral and adventitious roots requires cell wall degradation and assembly, as well as a fine-tuned crosstalk between microtubules and cell walls, auxin transport is required for the proper induction of the adventitious roots in *Arabidopsis* (da Costa et al., 2013; Abu-Abied et al., 2015a; Vilches-Barro and Maizel, 2015). However, our knowledge about the function and the regulatory processes controlling cell wall modifications during the early stages of adventitious root formation, even before resuming active cell divisions, is still limited. Cell wall integrity signaling is used by distantly related organisms to adapt the cell walls during growth and development (Wolf et al., 2012). The impairment of cell wall integrity affects plant developmental processes in response to mechanical modifications of cell walls, including cell-cycle progression, cell expansion and organogenesis (Landrein et al., 2015; Gigli-Bisceglia and Hamann, 2018; Vogler et al., 2019). Cell wall integrity signaling involves several components and pathways, including receptor-like kinases (RLKs), ion fluxes, RAC/ROP guanosine triphosphate (GTP)-binding proteins or cytoskeleton remodeling, which may control the auxin distribution (Sassi and Traas, 2015). The RLKs, including lectin-type RLKs (LecRLKs), leucine-rich repeat RLKs (LRR-RLKs), wall-associated kinases (WAKs) and *Catharanthus roseus* RLK1-like kinases (CrRLK1Ls), play significant roles in the signal transduction of cell wall-related signals. However, other cell wall and plasma membrane-localized proteins may also play roles in cell wall signaling, such as stretch-activated ion channels or arabinogalactan proteins (Wolf, 2017; Franck et al., 2018). Plant RAC/ROP GTP-binding proteins are membrane-associated proteins involved in many signaling processes, and specifically, their roles during polar growth are recognized as fundamental mechanisms involved in cell polarity (Qin and Dong, 2015). Plant RAC/ROP GTP-binding proteins may also play roles in transducing cell wall-related signals because specific members of this family act downstream of RLKs, especially CrRLK1Ls (Nibau and Cheung, 2011; Nissen et al., 2016). ROP signaling is also involved in the regulation of cytoskeleton reorganization, auxin carrier polarity and auxin distribution (Sassi et al., 2014).

A role for the interaction between cell wall–plasma membrane and cytoskeleton has been proposed as being involved in the maturation-related decline of adventitious root formation in pine (Pizarro and Díaz-Sala, 2019; Díaz-Sala, 2020). Signaling modulated by a dynamic extracellular matrix, along with soluble factors, plays important roles in remodeling morphogenesis in animal systems (Daley and Yamada, 2013). Specific and dynamic changes in the interactions between the cell wall and cytoskeleton, affecting the polarity of auxin efflux carriers in rooting progenitor cells prior to and after adventitious root formation irreversible declines, may represent possible targets for the developmental, environmental, hormonal and epigenetic regulation of the maturation-related decline in adventitious root formation (Díaz-Sala, 2019; Pizarro and Díaz-Sala, 2020, Díaz-Sala, 2020).

The objectives of this work were to analyze the expression dynamics of genes encoding proteins involved in the cell wall–plasma membrane–cytoskeleton continuum, as well as auxin carriers, and to identify groups of genes that may be involved in the cellular dynamics associated with maturation-related competence to form adventitious roots. For that purpose, the very simple experimental system that is based on the different rooting capacity of hypocotyl and epicotyl cuttings from young seedlings of pine was used. Briefly, whereas hypocotyl cuttings from 21-day-old seedlings of *P. radiata* rapidly form adventitious roots synchronously, hypocotyl and epicotyl cuttings from 91-day-old seedlings do not root or root poorly. A continuous ring of mature and active cambium, and a complete ring of secondary xylem develop in non-competent hypocotyls and epicotyls from 91-day-old seedlings, with interruptions at the primary leaf-axillary bud traces in epicotyls. However, while the cambium starts to form, it does not become differentiated or active in competent hypocotyls from 21-day-old seedlings. The system takes advantage of the different regeneration capacity of cambial and cambial-derived cell types at different developmental stages, and the absence of callus formation during the regeneration process. Therefore, a direct and synchronized developmental switch, without passing through a developmentally non-identified callus cell, can be studied. Although, the early cellular responses to auxin are similar in rooting-competent and non-competent cuttings, the rapid cell division and reorientation of division planes preceding root meristem organization are only observed in a short period of time (6 days) in competent cells. The rooting response of these cuttings in both *P. taeda* and *P. radiata*, as well as the cellular and anatomical events during the time course of adventitious root formation have been previously described (Díaz-Sala et al., 1996; Greenwood et al., 2001; Sánchez et al., 2007; Solé et al., 2008; Abarca et al., 2014). In this manuscript, the expression dynamics of 70 genes encoding proteins belonging to the aforementioned group, and for four auxin carrier proteins, are reported. The results demonstrated that the expression levels of cell wall integrity sensors and the signal transduction modules involving small GTP-binding proteins are associated with the capacity to form adventitious roots prior to the resumption of cell division and during the rapid cell division that leads to the organization of an adventitious root meristem.

## Materials and Methods

### Plant Material, Root Induction and RNA Extraction

Pine (*P. radiata* D. Don) seeds were germinated, and seedlings were grown as previously described (Sánchez et al., 2007). The seedlings were watered daily with water, and, after 21 days, weekly with 2 g/l of a commercial soluble fertilizer [NPK 20-7-19 (w/w/w)]. Cuttings for adventitious root induction were prepared in accordance with Sánchez et al. (2007). Briefly, rooting-competent hypocotyl cuttings, including the intact epicotyl, from 21-day-old seedlings (cH21) and non-competent hypocotyl (ncH91) or epicotyl (ncE91) cuttings from 91-day-old pine seedlings were prepared by severing the hypocotyl or epicotyl at its base, trimming it to 2.5 cm from the cotyledons (hypocotyls) or from the apical bud (epicotyls). All but one apical tuft of needles were removed from the epicotyls to obtain a foliar surface similar to that of the hypocotyls. Root induction was conducted by exposing the cuttings to 10 μM indole-3-butyric-acid (IBA) continuously. The IBA was obtained from Sigma (St. Louis, MO, United States) as IBA-K and dissolved in distilled water. For the analysis of gene expression during adventitious rooting, 50 basal segments, 1 cm in length, of the hypocotyl or epicotyl cuttings were pooled for each treatment at time 0 (time of excision) and after 1, 2, and 6 days of IBA treatment. Basal segments from 50 hypocotyl or epicotyl cuttings maintained in distilled water were also collected at the same time points and used as controls (mock). Basal segments from treated and non-treated cuttings were immediately frozen in liquid nitrogen and stored at −70°C until used for RNA isolation. Protocols for total RNA isolation and quantification from cuttings have been previously described (Sánchez et al., 2007). Total RNA was extracted using an RNeasy^®^ Plant mini kit (Qiagen GmbH), following the manufacturer’s instructions. The RNA concentration and quality were determined using a ND-1000 Spectrophotometer (NanoDrop Technologies Inc., United States). RNA was prepared from three independent biological replicates.

### NanoString Analysis

Sequences of 84 genes from *Pinus* sp. gene collections available in our laboratory were used as query probes to separately screen *P. radiata*, *P. taeda*, and *Pinus pinaster* transcriptome databases and GenBank using BLASTn. Sequences from these species with maximum homology levels (sup. 96%) to query probes were selected for probe design after confirming that the retrieved sequences represented genes encoding the same protein (Supplementary Table 1). Various members of the same multigene family were selected in cases of very close homology levels. The sequences were checked and used for custom CodeSet design to analyze expression levels during adventitious root formation. The NanoString CodeSet was designed and synthesized by NanoString Technologies (Supplementary Table 1). The CodeSet included 70 genes encoding proteins involved in the cell wall–plasma membrane–cytoskeleton continuum, four genes encoding auxin carriers, seven *P. radiata GRAS* genes of known expression during the adventitious root formation in the hypocotyl-epicotyl system (Abarca et al., 2014), which were used as references to ensure the accuracy of the technique in each experiment, and three genes encoding other proteins for their possible use as housekeeping genes. RNA samples (100 ng) were hybridized for 18 h with gene-specific color-coded probes, and data acquisition was carried out using an nCounter Digital Analyzer following the manufacturer’s instructions (NanoString Technologies) (Geiss et al., 2008). Normalization, differential expression and analyses of NanoString data were performed using nSolver Analysis Software 4.0 with the NanoString Advanced Analysis Module 2.0 plugin.^1^ Six positive-control and six negative-control probes were used to generate a standard curve for normalization, and four pine-specific genes (*ARF1*, *RAN1B*, *pG4.73*, and *SEC34*) that spanned a range of counts were used for CodeSet content normalization. Positive control normalization and CodeSet normalization were performed using means and geometric means to compute normalization factors, respectively, in accordance with NanoString recommendations. All the pairwise ratios were built for fold-change estimation. The false discovery rate (FDR) *p*-value adjustment was performed using the Benjamini–Yekutieli method (Benjamini and Yekutieli, 2001). Clustering was performed with the bottom-up approach of hierarchical agglomerative clustering using a Pearson’s correlation distance metric in which the distance between two clusters was calculated as the mean distance between their elements, as provided in the software. Z-score transformation (Z-score genes) was used for the heat map construction. A volcano plot was used to represent the linear regression of the differential gene expression for each variable. A principal component analysis (PCA) was performed to determine the impact of the expression levels of specific probes on the clustering of samples by contrasting principal components in pairs, as shown in biplots.

## Results

### Overall Analysis of the Cell Wall–Plasma Membrane–Cytoskeleton Gene Expression Response of Rooting-Competent and Non-competent Stem Cuttings During Adventitious Root Formation

**FIGURE 1:**
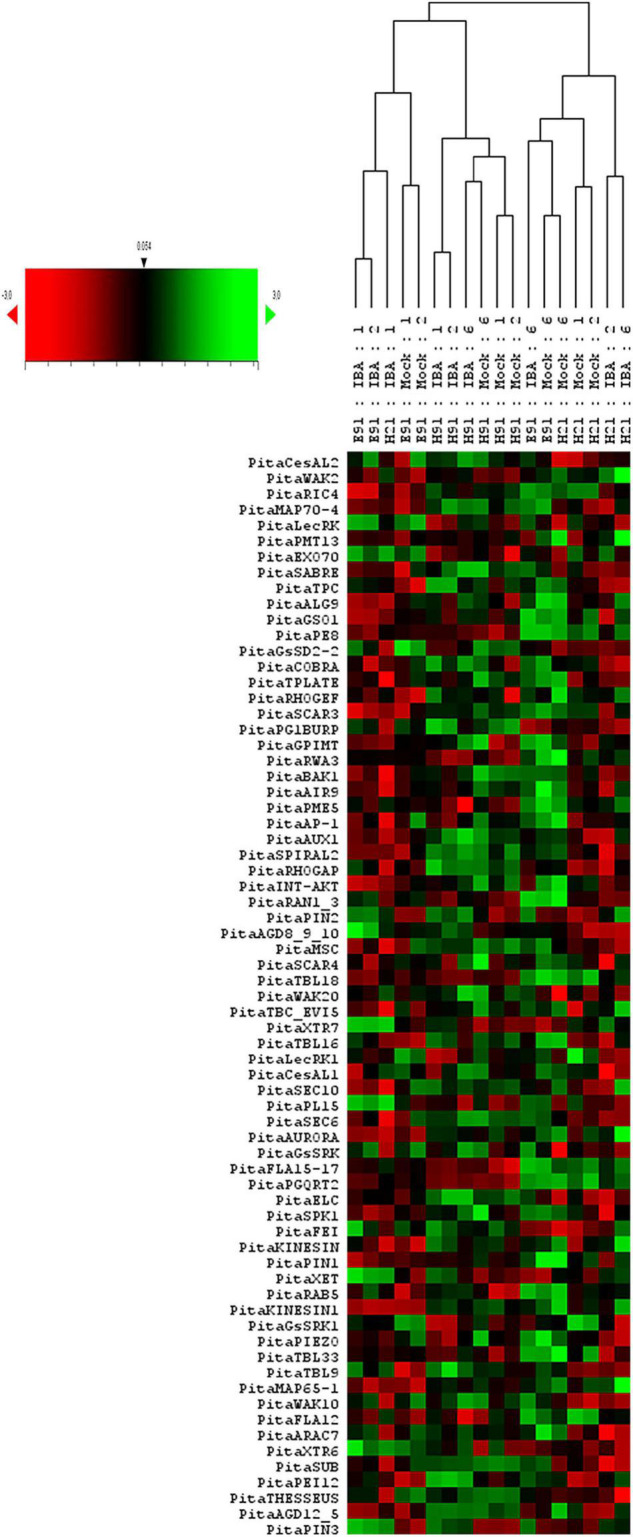
Expression profile of genes involved in the cell wall–plasma membrane–cytoskeleton continuum in rooting-competent hypocotyl cuttings from 21-day-old seedlings (H21) and in both non-competent hypocotyl (H91) and epicotyl (E91) cuttings from 91-day-old-seedlings during adventitious root formation. RNA was extracted from the base of hypocotyl (H) and epicotyl (E) cuttings treated with 10 μM indole-3-butyric-acid at the indicated times (days). Hypocotyl and epicotyl cuttings maintained in water were used as controls (mock). An agglomerative cluster analysis was performed based on the Pearson’s distance between gene expression levels and plotted as a heat map with a dendrogram of sample data.

To analyze the overall gene expression of rooting-competent and non-competent cuttings during development and in response to auxin during adventitious root formation, a hierarchical clustering based on Pearson’s distances and a PCA of gene expression levels in cuttings having different rooting capacities were performed at the time of excision and during adventitious root formation in response to auxin (Figure 1 and Supplementary Figures 1A,B, 2). An analysis of gene expression in cuttings at the time of excision showed the gene expression profiles of samples clustering in according with age and rooting capacity (Supplementary Figures 1A,B). Specific samples from ncE91 showed a gene expression close to that of cH21. Gene expression levels also differed among cH21, ncH91 and ncE91 over time during adventitious root formation (Figure 1 and Supplementary Figure 2). Two major sample clusters were defined on the basis of their gene expression profiles (Figure 1): (1) cH21 grouped separately from ncH91 and ncE91, except cH21-IBA, which grouped closely to ncE91-IBA, during the early stages of adventitious root formation; and (2) ncH91 and ncE91, except ncE91-mock and ncE91-IBA, which clustered closely to cH21-mock, at 6 days after excision. Two subclusters were defined within each cluster. Gene expression responses of cH21-IBA over 2 and 6 days, during which time adventitious root formation was induced, grouped separately from the responses of cH21-mock, as did ncE91 at 6 days after excision, which did not root. ncH91 clustered separately from ncE91 and from cH21-IBA. Although gene expression is affected by auxin in the three types of cuttings, because gene expression levels in non-treated hypocotyl or epicotyl cuttings clustered separately from gene expression levels in IBA-treated cuttings, cH21 and ncH91 clustered separately (Supplementary Figure 2). Gene expression levels from ncE91 clustered close to those of from ncH91. However, specific samples from ncE91 showed a gene expression pattern closer to that of cH21 (Supplementary Figure 2). Gene expression differences did not clearly differentiate among treatments or the time from excision (Figure 1 and Supplementary Figure 2).

### Expression Profiling Allows the Identification of Specific Differences in the Expression Patterns of Cell Wall–Plasma Membrane–Cytoskeleton Genes in Rooting-Competent and Non-competent Stem Cuttings During Adventitious Root Formation

**FIGURE 2:**
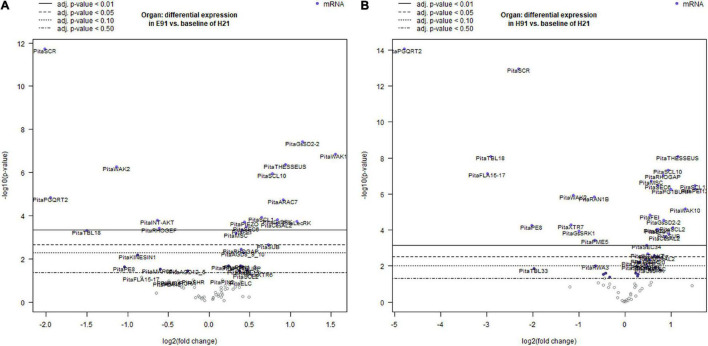
Volcano plots of a linear regression of the differential gene expression in epicotyl **(A)** and hypocotyl **(B)** cuttings from 91-day-old seedlings (E91 and H91, respectively) during adventitious root formation using the gene expression in hypocotyl cuttings from 21-day-old seedlings (H21) as the baseline. Point colors and horizontal lines indicate various false discovery rate (FDR) thresholds.

To characterize the dynamic patterns of gene expression during adventitious root formation in stem cuttings having different rooting capacities, an overall gene expression analysis was performed at the time of excision and during adventitious root formation in both cuttings. For the non-competent vs. competent cutting comparison, the expression levels in the competent cuttings served as the baseline. For treated vs. non-treated cutting comparison, the expression levels in the non-treated cuttings served as the baseline, and in each time point vs. time 0 (time of excision) comparisons, the expression at time 0 served as the baseline during adventitious root formation (Figures 2A,B; Supplementary Figures 3A,B, 4A–D; and Supplementary Table 2). Genes showing significant differential expression values in non-competent cuttings, using the expression of competent cuttings as the baseline at the time of excision, are shown in Supplementary Figures 3A,B and Supplementary Table 2. The number of differentially expressed genes was higher in ncH91 than in ncE91. The expression levels of several genes, most prominently genes encoding specific members of the TRICHOME BIRREFRINGENCE-LIKE (TBL) or FASCICLIN family, the G-type LecRLK GsSRK1, the potassium ion channel INT-AKT, the MICROTUBULE-ASSOCIATED PROTEIN SPIRAL2 and AUXIN-EFFLUX CARRIER PIN-FORMED1 (PIN1), significantly decreased in ncH91 and ncE91. The expression levels of *GsSRK*, *WAK20*, and *PIN3* significantly increased in both types of cuttings. Other genes encoding proteins involved in cell wall remodeling, RLKs, ion channels, cell wall-membrane nexus or cytoskeleton were also differentially expressed in either cutting type. Groups of genes encoding small GTP-binding-related proteins only differentially decreased in ncH91. Genes showing a significant differential expression value during adventitious root formation in non-competent cuttings, using the expression of competent cuttings as the baseline, are shown in Figures 2A,B and Supplementary Table 2. The number of differentially expressed genes was higher in ncH91 than in ncE91. Common and cutting-specific genes were differentially expressed in both cutting types. The expression levels of several genes were significantly decreased in ncH91 and ncE91, including, most prominently, those encoding members of the TBL family of proteins, POLYGALACTURONASE QRT2 ISOFORM (PGQRT2), WAK2 and the Rho-GTP-binding protein RHO GUANYL-NUCLEOTIDE EXCHANGE FACTOR 1 RHOGEF. The expression levels of genes in the RLK group, the G-type LecRLKs *GsSD2-2* and *GsSRK*, *WAK10*, the CrRLK1L *THESEUS*, the LRR-RLK *FEI* and mechanosensitive ion channels (*MSC*) significantly increased in both types of cuttings, as did genes encoding specific isoforms of cellulose synthase-like (CeSAL1), BURP domain-containing polygalacturonase 1, the microtubule-associated protein *AIR9*, the small GTP-binding proteins RAC GTP-BINDING PROTEIN ARAC7 and RHO GTPASE ACTIVATING PROTEIN RHOGAP, EXOCYST COMPLEX COMPONENT SEC6 and polar auxin transport (PIN2) proteins. Other genes encoding proteins involved in cell wall remodeling, RLKs, ion channels, cell wall-membrane nexus, small GTP-binding proteins or cytoskeleton were also differentially expressed specifically in one cutting type. The overall gene expression analysis in response to auxin showed that the number differentially expressed genes, compared with control, was less than those differentially expressed in cuttings, indicating the influence of the type of cutting on the gene expression response (Supplementary Figure 4A and Supplementary Table 2). Genes induced in the presence of exogenous auxin were mostly involved in the cell wall remodeling (XYLOGLUCAN ENDOTRANSGLYCOSYLASE *XTR7*, PECTINESTERASE INHIBITOR *PEI12*, ENDOXYLOGLUCAN GLYCOSYLTRANSFERASE *XET*, XYLOGLUCAN ENDO-4-BETA-D-GLUCANASE *XTR6*, PECTATE LYASE *PL15*, CELLULOSE SYNTHASE-LIKE PROTEIN D3, *CesAL2* and *TBL9*), RLK (*WAK2* and *FEI*) small GTP-binding proteins-related (RAB GTP-BINDING PROTEIN *RAB5*, *RHOGEF* and ADP-RIBOSYLATION FACTOR GTPASE-ACTIVATING PROTEIN *AGD8_9_10*) and polar auxin transport (*PIN2* and *PIN3*). The major reprogramming of gene expression occurred during the initial 48 h of the process (Supplementary Figures 4B–D and Supplementary Table 2). Most differentially expressed genes were downregulated during the time course, except specific genes of the cell wall remodeling (*XTR7*, *XTR6*, and *PGQRT2*) and RLK (*LecRK1*, *LecRK*, *GsSRK*, *GsSRK1*, *WAK10*, *WAK20*, *THESSEUS*, and *GsSD2-2*) groups. The mechanosensitive channel PIEZO and genes included in the membrane trafficking group (AP-1 COMPLEX SUBUNIT and EXOCYST COMPLEX COMPONENTS) were differentially upregulated at 6 days after induction.

### Dynamic Gene Expression Patterns During Adventitious Root Formation in Stem Cuttings Having Different Rooting Capacities

The genes showing the largest variations in expression levels (≤−0.85 Log_2_FC and ≥0.85 Log_2_FC) were selected for further analyses (Supplementary Table 2). This set of genes was divided in five categories on the basis of their functions, as follows: cell wall modification-related genes, RLKs, small GTPase-related genes, microtubule- and microtubule organization-associated genes and auxin transport protein-encoding genes. Ion channel-related genes, including mechanosensitive ion-channels, were also included in the analysis because of their functional significance.

### Expression Profiles of Genes Encoding Proteins Involved in Cell Wall Modification

**FIGURE 3:**
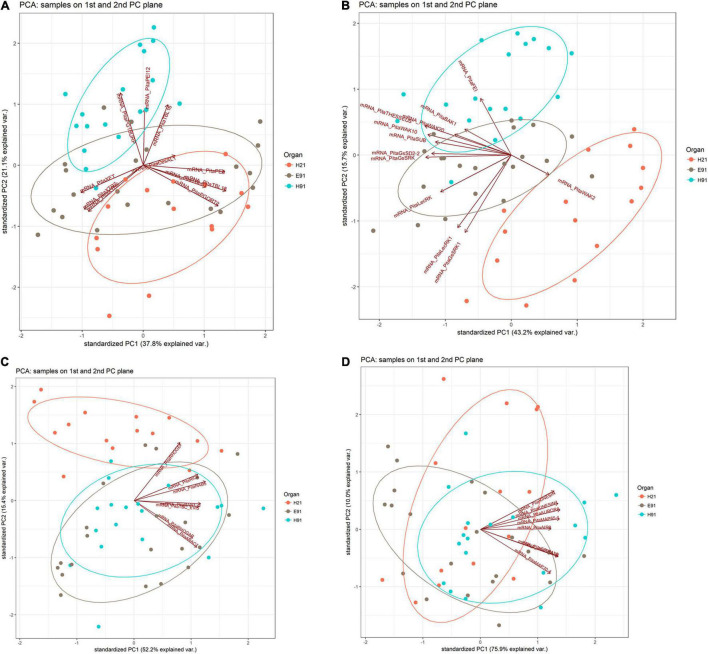
Principal component analysis-biplot showing the expression distribution of genes encoding cell wall modification **(A)**, receptor-like kinases **(B)**, small GTPase-related **(C)**, and cytoskeleton-related **(D)** proteins in rooting-competent hypocotyl cuttings from 21-day-old seedlings (H21) and both non-competent hypocotyl (H91) and epicotyl (E91) cuttings from 91-day-old-seedlings in a two-dimension surface extracted from the principal component analysis.

**FIGURE 4:**
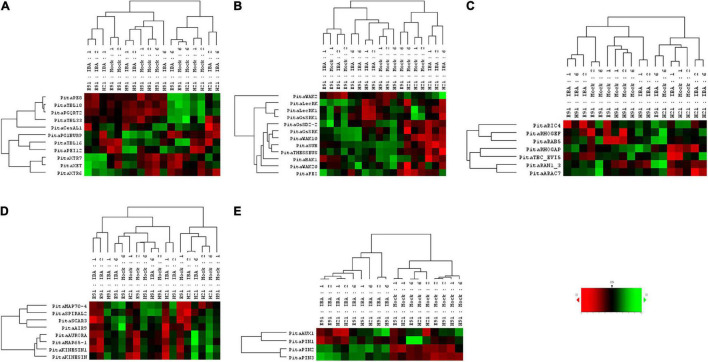
Expression profile of genes encoding cell wall modification **(A)**, receptor-like kinases **(B)**, small GTPase-related **(C)**, cytoskeleton-related **(D)**, and auxin carrier **(E)** proteins in rooting-competent hypocotyl cuttings from 21-day-old seedlings (H21) and both non-competent hypocotyl (H91) and epicotyl (E91) cuttings from 91-day-old-seedlings during adventitious root formation. RNA was extracted from the bases of hypocotyl (H) and epicotyl (E) cuttings treated with 10 μM indole-3-butyric-acid at the indicated times (days). Hypocotyl and epicotyl cuttings maintained in water were used as controls (mock). An agglomerative cluster analysis was performed based on Pearson’s distances between gene expression levels and plotted as a heat map with a dendrogram of sample and gene data.

The expression patterns of genes encoding 11 proteins related to hemicellulose and pectin metabolism shared a close relationship between cH21 and ncH91 at the time of excision (Supplementary Figure 5A). A high number of genes, including genes involved in both hemicellulose and pectin metabolism, but mostly involved in hemicellulose remodeling or modification, were upregulated in cH21 at the time of excision. The expression levels of the genes encoding other isoforms were intermediate between those of ncH91 and ncE91. Overall, genes encoding proteins involved in hemicellulose and pectin metabolism were mostly expressed in an auxin-, age-, or developmental-dependent manner during adventitious root formation. Organ and age seemed to influence the expression of this group of genes to a higher extent than auxin exposure or time of induction (Figure 3A and Supplementary Figures 6A, 7A). However, the gene expression responses of cH21 and ncE91 were closely related compared with that of ncH91 (Figure 3A), during the early and late stages of adventitious root formation in non-treated and treated cuttings (Figure 4A). Two major clusters of samples were defined on the basis of their expression profiles (Figure 4A): (1) cH21 grouped separately from ncH91 and ncE91, except cH21-IBA, which grouped closely to ncE91-IBA, during the early stages of adventitious root formation; and (2) ncH91 and ncE91, except ncE91-mock and ncE91-IBA, which clustered closely to cH21-mock, at 6 days after excision. Two subclusters were defined within each cluster. The responses of cH21-IBA over 2 and 6 days, during which time adventitious root formation was induced, grouped separately from the responses of cH21-mock, as well as ncE91 at 6 days after excision. They did not induce adventitious rooting. ncH91 clustered separately from ncE91 during the initial 2 days of the root induction process, and from cH21-IBA, which grouped closely to ncE91-IBA during the early stages of adventitious root formation. Auxin affected gene expression patterns in the three types of cuttings because non-treated cuttings clustered separately from IBA-treated cuttings. Three major expression patterns were defined on the basis of the hierarchical clustering (Figure 4A): (1) Genes with higher expression levels in cH21 under control conditions and in the presence of IBA, as well in ncE91 after 6 days. Expression levels were lower in the presence of IBA. This cluster was mainly enriched with *PE8*, *TBL18*, and *PGQRT2*; (2) Genes with mostly downregulated expression levels in cH21-IBA during the early or late stages of adventitious root formation. In ncH91 and ncE91, expression levels were higher than in cH21. This cluster was mainly enriched with *TBL33*, *CeSAL1*, BURP domain-containing polygalacturonase 1 and *TBL16*; and (3) Genes, mostly in cH21 and ncE91, with auxin-induced expression during the early stages of adventitious root formation. This cluster was mainly enriched with *PEI12*, *XET*, *XTR6*, and *XTR7*. A biplot analysis based on the first two components of a PCA (Figure 3A) showed that the expression levels of *PECTIN ESTERASE PE8*, *TBL18*, *TBL33*, and *PGQRT2* were strongly positively correlated to each other and showed weaker correlations to the expression levels of *XET*, *XTR6*, and *XTR7*, which were strongly positively correlated to each other, as well as to *CeSAL1* and *TBL16*. The expression levels of *PGBURP* and *PEI12*, which were strongly positively correlated to each other, had strong correlations to the expression level of *TBL16*, weak or no correlation with the expression level of *CeSAL1*, and were strongly negatively correlated to the expression levels of *PE8*, *TBL8*, *TBL33*, *PGQRT2*, *XET*, *XTR6*, and *XTR7*. The expression level of *CeSAl1* showed a negative correlation with the expression levels of *XET*, *XTR6*, and *XTR7*.

### Expression Profiles of Genes Encoding Receptor-Like Kinases and Ion Channels

**FIGURE 5:**
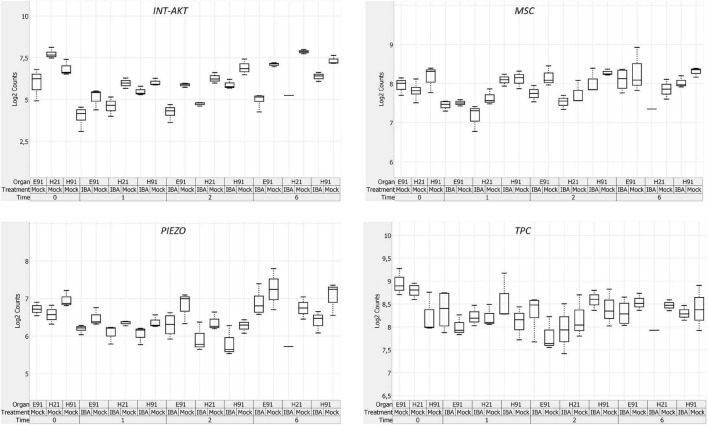
Expression profile of genes encoding ion channels in rooting-competent hypocotyl cuttings from 21-day-old seedlings (H21) and both non-competent hypocotyl (H91) and epicotyl (E91) cuttings from 91-day-old-seedlings at the time of excision (t0) and during adventitious root formation. RNA was extracted from the bases of hypocotyl (H) and epicotyl (E) cuttings treated with 10 μM indole-3-butyric-acid at the indicated times (days). Hypocotyl and epicotyl cuttings maintained in water were used as controls (mock). A box plot analysis was performed on the basis of the NanoString results. The bottom and top of each box indicates the 25th and 75th percentile of data expression values, respectively, the band in the middle of the box indicates the median expression value, and the ends of the whiskers indicate the minimum and maximum values. Expression values for the box-plot analysis were log2 transformed.

The expression patterns of genes encoding 11 RLKs belonging to four different families of proteins, LecRLKs (L- and G-types), WAKs, CrRLK1Ls, and LRR-RLKs, were closely related among non-competent cuttings at the time of excision (Supplementary Figure 5B). *LecRLKs*, *WAK10*, *WAK20*, *GsSRK*, *GsSD2-2*, and *THESEUS* showed the lowest expression levels in cH21, whereas *LRR-RLK BAK1*, *GsSRK1*, and *WAK2* showed the highest levels. *FEI* and the *LRR-RLK STRUBBELIG-RECEPTOR SUB* showed intermediate levels. The gene expression levels in this group differed between hypocotyl and epicotyl cuttings during adventitious root formation. The expression of these genes was more affected by organ and age than by auxin and time of induction (Figure 3B and Supplementary Figures 6B, 7B). Two major clusters of samples were defined on the basis of their expression profiles (Figure 4B): (1) cH21 grouped separately from ncH91 and ncE91, except ncE91-mock at 6 days after excision; and (2) ncH91 and ncE91. Two subclusters were defined within each cluster. cH21-mock, including ncE91-mock after 6 days of excision, which did not root, grouped separately from cH21-IBA, which induced adventitious root formation. Similarly, ncH91-mock and ncE91-mock clustered separately from ncH91-IBA and ncE91-IBA, respectively. In addition, the gene expression responses of ncE91 during the initial 2 days of the root induction process differed from the ncH91 responses, tending to closely respond after 6 days of exogenous auxin treatment. Three major expression patterns were defined on the basis of the hierarchical clustering (Figure 4B): (1) Genes with expression levels that decreased in cH21-IBA after 6 days. This cluster was mainly enriched with L-type *LecRLK*s and G-type *GsSRK1* genes; (2) Genes with lower expression levels in cH21 than in ncH91 and ncE91 during root induction, especially after 6 days. The expression levels of these genes were not affected or decreased in IBA-treated cuttings. This cluster was enriched with *THESEUS*, *GsSD2-2*, *GsSRK*, *WAK10*, *WAK20*, *BAK1*, and *SUB*. *FERONIA* mRNA was detected at below the background level (data not shown); and (3) *FEI* and *WAK2* expression levels increased in IBA-treated cuttings, especially after 6 days of treatment. A biplot analysis based on the first two components of a PCA (Figure 3B) showed that the expression levels of *GsSRK*, *GsSD2-2*, *THESESUS*, *WAK10*, *WAK20*, *SUB*, and *BAK1* were strongly positively correlated to each other and showed weaker correlations with the expression levels of *GsSRK1*, *LecRK*, *LecRK1*, and *FEI*, whereas the expression of *WAK2* was strongly negatively correlated, or showed no correlation, with the expression levels of other RLKs. The expression levels of genes encoding two mechanosensitive channels MSC and PIEZO, the potassium channels TPC and INT-AKT were also analyzed (Figure 5). *MSC* was downregulated in the presence of auxin in cH21 from the early stages of adventitious root formation, whereas *PIEZO* and *TPC* were downregulated at 6 days. *INT-AKT* was downregulated in the presence of auxin in cH21 and ncE91 mainly from the early stages of adventitious root formation.

### Expression Profiles of Genes Encoding Small GTP-Binding Proteins

The expression patterns of genes encoding five small GTP-binding proteins, belonging to the Rho (Rop), Rab, and Ran groups, a downstream effector of the Rho-related GTP-binding proteins, the CRIB domain-containing protein RIC4 and the Rab-GAP TBC domain-containing protein EVI5 TBC_EVI5, were closely related between cH21 and ncH91 at the time of excision (Supplementary Figure 5C). All the genes showed higher expression levels in cH21. *RIC4*, *RHOGEF*, and *RAB5* showed lower expression levels in ncE91. *RHOGAP*, *RAN GTP-BINDING PROTEIN RAN1_3*, *TBC_EVI5*, and *ARAC7* showed lower expression levels in ncH91. The gene expression levels of this group also differed between hypocotyl and epicotyl cuttings during adventitious root formation. The expression of these genes was more affected by organ and age than by auxin and time of induction (Figure 3C and Supplementary Figures 6C, 7C). Two major clusters of samples were defined on the basis of their expression profiles (Figure 4C): (1) cH21 grouped separately from ncH91 and ncE91, except ncE91-IBA after 6 days of excision; and (2) ncH91 and ncE91. Two subclusters were defined within each cluster. The responses of cH21 during the initial 24 h grouped separately from the responses over 2 and 6 days, including ncE91-IBA at 6 days after excision. cH21-IBA, which induced adventitious root formation, grouped separately from cH21-mock at day 2 and 6. Similarly, ncH91-IBA and ncE91-IBA clustered separately from ncH91-mock and ncE91-mock, respectively. In addition, the gene expression responses of ncE91 during the initial 2 days of the root induction process differed from the ncH91 responses, tending to maintain a separation after 6 days of exogenous auxin treatment. Two major expression patterns were defined on the basis of hierarchical clustering (Figure 4C): (1) Genes with downregulated expression levels in cH21-IBA at 6 days after induction, but expression levels were similar or higher than those in ncH91 and ncE91. This cluster was mainly enriched with *RIC4*, *RHOGEF*, and *RAB5*. *TBC_EVI5* was downregulated during the early stages of adventitious root formation in cH21. After 6 days, a significant increase in the expression levels was measured; and (2) Genes with downregulated expression levels in cH21-IBA either from the early stages or after 6 days. In ncH91 and ncE91, expression levels were higher than in cH21, either from the initial stages of the rooting process or after 6 days. This cluster was mainly enriched with *ARAC7*, *RAN1_3*, and *RHOGAP*. The expression levels of these genes were not affected or were they decreased in IBA-treated cuttings. A biplot analysis based on the first two components of a PCA (Figure 3C) showed that the expression levels of *RAN1_3* and *TBC-EVI5* were strongly positively correlated with each other and showed strong correlations with the expression levels of *RIC4* and *RAB5*, which were strongly positively correlated with each other, as well as with *RHOGAP* and *ARAC7*, which were also strongly positively correlated with each other, but had a weaker correlation with the expression level of *RHOGEF*. *RHOGEF* showed a positive correlation with the expression levels of RIC4 and *RAB5* and no correlation with the expression levels of *RHOGAP* and *ARAC7*. *RIC4* and *RAB5* showed weak positive correlations with the expression levels of *RHOGAP* and *ARAC7*.

### Expression Profiles of Genes Encoding Cytoskeleton-Related Proteins

The expression patterns of genes encoding eight microtubule- and microfilament-associated proteins involved in cytoskeleton organization, mostly during cell division and expansion, were closely related between cH21 and ncE91 at the time of excision (Supplementary Figure 5D). *SPIRAL2*, MICROTUBULE-ASSOCIATED PROTEIN *MAP65-1*, SERINE THREONINE-PROTEIN KINASE *AURORA*, *AIR9* and PHRAGMOPLAST-ASSOCIATED KINESIN-RELATED PROTEIN (*KINESIN*) showed the highest expression levels in cH21 at the time of excision, whereas MICROTUBULE-ASSOCIATED PROTEIN *MAP70-4*, SCAR/WAVE PROTEIN *SCAR3* and PHRAGMOPLAST-ORIENTING KINESIN (*KINESIN1*) showed the highest levels in ncH91. The expression levels in cH21 were lower than in ncH91 and higher than in ncE91, which showed the lowest expression levels. Overall, genes encoding proteins involved in microtubule- and microtubule organization-associated proteins were mostly expressed in an auxin-, age-, or developmental-dependent manner during adventitious root formation. Auxin and time of the induction affected the expression levels of these groups of genes to a slightly higher extent than organ and age (Figure 3D and Supplementary Figures 6D, 7D). The responses of cH21-IBA after 2 days were grouped separately. Two major expression patterns were defined on the basis of hierarchical clustering during adventitious root formation (Figure 4D): (1) Genes with downregulated expression levels in cH21-IBA, either from the early stages or after 6 days. In ncH91 and ncE91, expression levels were higher than in cH21, either from the initial stages of the rooting process or after 6 days. This cluster was mainly enriched with *SPIRAL2*, *SCAR3*, *MAP70-4*, and *AIR9*. Expression levels of these genes were not affected, or were decreased, in IBA-treated cuttings; and (2) Genes with similar or higher expression levels in cH21-IBA than in ncH91-IBA and ncE91-IBA after 2 days. This cluster was mainly enriched with *AURORA*, *KINESIN*, *MAP65-1*, and *KINESIN1*. The expression levels of these genes were not affected, or were decreased, in IBA-treated cuttings. All the genes were downregulated in cH21-IBA and ncE91-IBA during the early stages of adventitious root formation. A biplot analysis based on the first two components of a PCA (Figure 3D) showed that the expression levels of *KINESIN*, *KINESIN1*, *AURORA*, *MAP65-1*, and *AIR9* were strongly positively correlated with each other and showed weaker correlations with the expression levels of *SPIRAL2*, *SCAR3*, and *MAP70-4*, which were also strongly positively correlated with each other.

### Expression Profiles of Genes Encoding Polar Auxin Transport Proteins

The expression patterns of genes encoding four proteins involved in polar auxin transport belonging to the AUX/LAX and PIN families were closely related between ncH91 and ncE91 at the time of excision (Supplementary Figure 5E). The expression level of *PIN1* was higher in cH21, whereas the *AUX1* expression level was intermediate and the *PIN2* and *PIN3* levels were lower. Auxin was the major factor affecting their expression during adventitious root formation (Figure 4E). Overall, these genes were expressed mostly in an auxin-dependent manner during adventitious root formation. The responses of cH21 and ncE91 were closer than the responses of ncH91 (Figure 4E). Two major expression patterns were defined on the basis of hierarchical clustering during adventitious root formation (Figure 4E). (1) Genes with downregulated expression levels in cH21-IBA and ncE91-IBA at the early stages of adventitious root formation. This cluster was mainly enriched with *AUX1* and *PIN1*; and (2) Genes with higher expression levels in cH21-IBA, ncH91-IBA, and ncE91-IBA during adventitious root formation. This cluster was mainly enriched with *PIN2* and *PIN3*.

## Discussion

The recalcitrance of stem cuttings to adventitious root formation is a major limitation in the clonal propagation or micropropagation of elite germplasms of many forest tree species, especially at the mature stage (Díaz-Sala, 2016). Several interrelated pathways may be involved in the plasticity required by plant cells for the regeneration of forest tree species (Bonga, 2016). Changes in the physical properties of the cell or tissue resulting in the modification of the cell polarity and the mechanical or physical aspects underlying modifications of cell division planes have been proposed as possible target pathways for the developmental, environmental, hormonal and epigenetic regulation of the maturation-related decline of adventitious root formation in pine (Pizarro and Díaz-Sala, 2020; Díaz-Sala, 2020).

### Expression Patterns of Genes Encoding Proteins Involved in the Cell Wall–Plasma Membrane–Cytoskeleton Continuum Are Associated With Organ and Age in Rooting-Competent and Non-competent Stem Cuttings During Adventitious Root Formation

A hierarchical clustering and PCA analysis of the cell wall–plasma membrane–cytoskeleton gene expression patterns in cuttings having different rooting capacities revealed a close relationship among non-competent cuttings at the time of excision (Supplementary Figure 1). Although both types of cuttings have different developmental origins, this result may indicate a closer developmental stage than that of rooting-competent cuttings in terms of the processes involving the analyzed genes. However, the higher number of responsive genes in ncH91 than in ncE91 at the time of excision and during adventitious root formation, as well as the specific expression in either cutting type (Figures 1, 2; Supplementary Figures 1–4; and Supplementary Table 2), suggests that developmental differences also exist among non-competent cuttings, because the lack of rooting competence is a common feature of both cutting types. Both cH21 and ncE91 showed primary structures and active primary growth. The cambium had begun to form in cH21 but was not developed at the rooting sites. In ncE91, the cambium was fully developed and the ring of xylem had begun to form. The cambium was also fully developed in ncH91. However, the continuous xylem ring is completely differentiated, and growth cessation has occurred (Díaz-Sala et al., 1996; Solé et al., 2008; Abarca et al., 2014). Organ and age influence the gene expression responses to wounding and auxin exposure more than phytohormone exposure or the rooting time course during adventitious root formation (Figure 1 and Supplementary Figure 2). The initial responses were similar between cH21-IBA and ncE91-IBA, and the late responses of ncE91 were similar to those of cH21-mock, which did not root. This indicated that the developmental relationship between cH21 and ncE91, and the overall gene expression pattern, were close to the rooting responses associated with the age and maturation of the cuttings. Although ncH91 and ncE91 may show different developmental stages, common expression patterns of specific genes involved in the cell wall–plasma membrane–cytoskeleton continuum, especially for the RLKs and GTP-binding-related protein groups at the time of excision and during adventitious root formation, indicated that both types of cuttings maintain common developmental pathways and that these pathways may be associated with the capacity to root (Figures 3, 4; Supplementary Figures 3–5; and Supplementary Table 2). Overall, these results may indicate the following: (1) the overall gene expression pattern was associated with the age and maturation of hypocotyl and epicotyl stem cuttings at the time of excision and in response to adventitious root induction; (2) these genes may have functions in adventitious rooting in cH21 from day 2 of treatment; and (3) the loss of the rooting competence of ncH91 and ncE91 may require different mechanisms regarding the expression patterns of genes involved in the cell wall–plasma membrane–cytoskeleton continuum, or both types of cuttings may show different developmental stages at the time of excision in which rooting competence has already been lost. Cambial cells showed active multiplicative division for maintaining the periclinal orientation of the division plane in rooting-competent and non-competent cuttings. At the time the cambial cells associated with the resin canals in competent cuttings, rapid and active formative divisions were associated with the modification of the orientation of the division plane, leading to the organization of an adventitious root meristem. The coordination of cell polarity, which is involved in cell wall modification, microtubule organization, membrane trafficking, auxin transport and distribution, and asymmetric cell division, resulting in the modification of cell division planes, regulates cell behavior in response to exogenous and endogenous developmental signals (Shao and Dong, 2016; Cascallares et al., 2020; Yang et al., 2020).

### Differential Expression of Genes Encoding Cell Wall Remodeling Proteins Is Both an Early and a Late Response of Rooting-Competent and Non-competent Stem Cuttings During Adventitious Root Formation

Cell wall properties and modifications affect adventitious root formation in plant species (Duman et al., 2020a; Li, 2021). Differentially expressed cell wall genes in hypocotyls and epicotyls at the time of excision and during adventitious root formation are functionally associated with pectin and hemicellulose metabolism, indicating the roles of cell wall remodeling in growth and the maturation-related decline of adventitious root formation in pine. Most genes showed high mRNA levels in cH21 and to a lesser extent in ncE91, indicating the high level of cell wall remodeling in both organs, especially in cH21, at the time of excision. However, mRNA levels were lower for most genes in ncH91 (Supplementary Figure 5A). The cell wall remodeling capacity may differ between organs displaying active growth and elongating cells, such as cH21 and ncE91, and non-growing organs with high rates of differentiated cells and growth cessation at the time of excision, such as ncH91 (Chebli and Geitmann, 2017). These genes are mainly expressed in organ- and age-dependent manners during adventitious root formation in response to wounding and auxin (Figure 3A and Supplementary Figures 6A, 7A). Although the initial response to IBA was closely related in the three cutting types, especially between cH21 and ncE91, the distance of the response of cH21-IBA after 2 days, and the separation between cH21 and ncH91, indicated that these genes may also be involved in the maturation-related decline of adventitious root formation after 48 h of treatment (Figures 3A, 4A). Overall, the results suggest that cell wall remodeling is both an early response, before resuming cell division, and a late response, after the induction of rapid cell division and meristem organization, to wounding and auxin in the three cutting types during adventitious root formation (Figure 4A). The differential expression of these genes may be related to specific mechanisms involved in the maintenance of cell wall integrity in each cutting (Hamann, 2015a). These mechanisms may be directly or indirectly relevant for adventitious root formation in competent cuttings. Key components involved in cell wall loosening and remodeling, such as expansins and both xyloglucan- and pectin-modifying enzymes, have been associated with adventitious root formation in several species, including tree species (Hutchison et al., 1999; Guenin et al., 2011; Rigal et al., 2012; Braybrook and Peaucelle, 2013; Lu et al., 2017; Sala et al., 2017; Lei et al., 2018; Li et al., 2018; Duman et al., 2020a,b; Eliyahu et al., 2020; Li, 2021). Although pectin methyl esterases and xyloglucan endotransglucosylases/hydrolases have already been associated with adventitious root formation (Guenin et al., 2011; Sala et al., 2017; Duman et al., 2020a,b), information on the roles of other polysaccharide modifications are still limited. Trichome birefringence-like proteins are involved in several processes related to cell wall biology, such as cellulose biosynthesis, the specific O-acetylation of cell wall polymers, bridging proteins that binds pectin and other cell wall polysaccharides, and the maintenance of pectin esterification (Bischoff et al., 2010a,b; Gille and Pauly, 2012; Stranne et al., 2018; Qaseem and Wu, 2020). The different expression patterns of different members of these families in rooting-competent and non-competent cuttings over the course of adventitious root formation, as well as the differences in the expression-related correlations of different members of the group (Figures 3A, 4A), may reflect the diversity of acetylated substrates and the specificity of TBL isoforms in the responses of various tissues at different developmental stages. Low mRNA levels of specific polygalacturonase isoforms (*PGBURP*), *TBL16*, and *CesAL1* genes in cH21-IBA or high mRNA levels of *PE*, *TBL18*, *PGQRT2*, and *TBL33* in cH21-mock and ncE91 at the late stage, which did not root, along with the lack of variation in ncH91, may indicate a requirement for modifications in cell wall softening/stiffening for root induction (Figure 4A). Adventitious rooting capacity has been associated with a decrease in cell wall stiffening, mediated by increases in xyloglucan endotransglucosylases/hydrolases and decreases in pectin methylesterase and polygalacturonase, in etiolated and green branches of avocado (Duman et al., 2020b). Variations in the expression levels of different pectin isoforms or xyloglucan-modifying enzymes may also indicate that their modification promotes wall stiffening or loosening, depending on the cutting, age, or the presence of auxin. However, the close responses between cH21 and ncE91 (Figure 4A) at specific times suggested that additional signaling pathways are involved in the regulation of adventitious rooting capacity.

### Expression Patterns of Genes Encoding Receptor-Like Kinases Are Correlated With Maturation-Related Decline of Adventitious Root Formation

Perturbations of cell wall integrity revealed sensing mechanisms to transduce cell wall impairment signals and maintain developmental homeostasis. RLKs and mechanosensitive channels in plants include a large family of proteins with potential cell surface signaling functions. These genes are mainly expressed in organ- and age-dependent manners during adventitious root formation in response to wounding and auxin (Figure 3B and Supplementary Figures 6B, 7B). Overall, the low mRNA level measured for most *RLK* and mechanosensitive channel genes (*MSC* and *PIEZO*) in cH21 at the time of excision and during adventitious root formation in response to IBA indicates a close association with the rooting capacity (Figures 3B, 4B, 5). Specific members of these families are involved in cell elongation, polarized growth, cell–cell communication, hormone regulation and cell wall synthesis, remodeling and sensing during both vegetative and reproductive development (Guo et al., 2009; Lin et al., 2012; Vaid et al., 2013; Engelsdorf and Hamann, 2014; Kohorn, 2015; Galindo-Trigo et al., 2016; Wolf, 2017; Franck et al., 2018; Schoenaers et al., 2018; Jose et al., 2020). Although, specific members of the *CrRLK1Ls*, *Lec*RLKs, or *SUB* are involved in lateral root formation and root development in model and forest tree species (Vaddepalli et al., 2017; Gonneau et al., 2018; Guo et al., 2019), there is no published data on their involvement in adventitious rooting and the maturation-related decline of adventitious root formation in conifers. Most genes showed high or intermediate mRNA levels in ncH91 and ncE91 compared with in cH21 (Supplementary Figure 5B) indicating functional roles, and perhaps, the redundancy of different RLKs, in processes involved in cell elongation and differentiation (Hamann, 2015a,b). The expression levels of these genes were associated with the rooting capacity during adventitious root formation in response to wounding and auxin (Figures 3B, 4B, 5). Although the initial response to IBA was similar for specific genes between rooting-competent and non-competent cuttings, the distance of the responses between rooting-competent and non-competent cuttings, and in their responses to auxin exposure, indicated that these genes may be involved in the maturation-related decline of adventitious root formation. Overall, these results suggested that most RLKs and specific mechanosensitive channels should be downregulated during adventitious root formation for root induction, perhaps to avoid specific functional redundancies and compensation involved in the maintenance of tissue and organ integrity (Banavar et al., 2021). An L-type LecRLK, LecRK-I.9, mediates cell wall–plasma membrane contacts through protein–protein interactions using arginine–glycine–aspartic acid (RGD)-containing proteins as the potential ligands (Bouwmeester et al., 2011). The RGD tripeptide motif is a well-known cell adhesion motif in mammalian cells (Ruoslahti, 1996). In plants, several studies have shown that exogenously applied RGD peptides have a strong effect on plant cells by disrupting cell wall–plasma membrane contacts (Canut et al., 1998). Interestingly, treating mature hypocotyls from *Arabidopsis*, which had lost their adventitious rooting capacity, with RGD peptides results in a recovery of the capacity to induce roots (Díaz-Sala et al., 2002). The downregulation of *LecRLK*s and other *RLK*s in cH21 may help maintain the competence to root by loosening the cell wall–plasma membrane signaling adhesion. Specific members of the WAK family physically interact with the wall (Decreux and Messiaen, 2005). Interestingly, this system is involved in cambial activity and xylem development in poplar (Song et al., 2011; Kumar et al., 2020). The upregulation of *WAK2* and *FEI* in response to auxin indicates that specific members of RLKs may regulate cell behavior positively in response to cell wall perturbations. The expression levels of specific Gossypium WAK genes significantly increase under auxin treatment (Dou et al., 2021). In addition, the disruption of the two partially redundant FEI1 and FEI2 RLKs causes root growth arrest (Basu et al., 2016).

### Expression Patterns of Genes Encoding Small GTP-Binding Proteins Are Correlated With Maturation-Related Decline of Adventitious Root Formation

Small GTP-binding proteins play pivotal roles in diverse cellular processes, such as RLK signal transduction, cytoskeletal organization, cell polarity, cell proliferation and differentiation, intracellular membrane trafficking and transport vesicle formation, and nucleocytoplasmic transport (Nielsen, 2020). Differentially expressed genes in hypocotyls and epicotyls at the time of excision and during adventitious root formation are functionally associated with Ran-, Rab-, and Rho-small GTP-binding proteins, indicating roles in endomembrane trafficking, RLK signal transduction and cytoskeleton dynamics, among others, in the growth and the maturation-related decline of adventitious root formation in pine. Most genes showed high mRNA levels in cH21, indicating high vesicle trafficking and cytoskeleton dynamics in an organ undergoing active growth and cell elongation at the time of excision. However, mRNA levels were lower for most genes in ncH91 and ncE91 (Supplementary Figure 5C). Although both cH21 and ncE91 showed active growth and elongating cells, the differential expression levels of vesicle trafficking- and cytoskeleton dynamic-related genes may indicate that their expression is associated with common processes in ncE91 and ncH91, with the latter showing a high rate of cell differentiation at the time of excision. These genes were expressed following organ- and age-dependent manners during adventitious root formation in response to wounding and auxin (Figure 3C and Supplementary Figures 6C, 7C). The distance of the response between rooting-competent and non-competent cuttings in response to auxin indicates that these genes may be related to the maturation-related decline of adventitious root formation (Figures 3C, 4C), highlighting the possible roles of GTP-binding proteins in the process. Members of this family are involved in several organogenesis processes in response to developmental and environmental signals, including lateral root formation and root development (Chen et al., 2011; Inoue et al., 2013; Nibau et al., 2013). The downregulation of specific *RHO*- and *RAN*-*GTPases*, as well as specific *RHO* regulatory factors involved in deactivation of GTPases occurred in cH21. The differential expression levels of these genes may be related to the downregulation of RLKs in the same cuttings caused by wounding and auxin treatments, because RAC/ROP GTPases have been described as downstream signaling elements of specific CrRLK1Ls, such as FERONIA and THESEUS (Cheung and Wu, 2011; Galindo-Trigo et al., 2016). The upregulation of *RAB*-*GTPases* and specific *RHO* regulatory factors involved in the activation of GTPases in cH21 during adventitious root formation may reflect the specificity of GTPase isoforms in the responses of various tissues at different developmental stages. A specific member of the *Arabidopsis* RAB GTPases, RAB-A5c, controls the growth direction during lateral root formation and organogenesis, interacting with cortical microtubules and cell wall mechanical properties (Kirchhelle et al., 2016, 2019). However, specific members of the *Arabidopsis* Rop-GEF family are involved in root patterning formation that connects RAC/ROP signaling, auxin-dependent *PLETHORA* expression and polar auxin transport (Chen et al., 2011). Information on the roles of GTPases in adventitious root formation are still limited. Members of the ADP-ribosylation factor GTPases, such as GNOM and ARF-GAP proteins, are involved in adventitious root formation (Zhuang et al., 2005; Liu et al., 2009). Perhaps all the genes have regulatory roles in the maturation-related decline in adventitious root formation in response to wounding and auxin, as well as in modifications in the cell wall and RLK signal transduction, but in diverse developmental and environmental contexts and tissues because each member of the GTPase gene family had its own unique expression pattern under wounding and auxin treatments.

### Cytoskeleton-Related Genes Are Expressed Following an Auxin-, Age-, or Developmental-Dependent Manner in Rooting-Competent and Non-competent Stem Cuttings During Adventitious Root Formation

The cytoskeleton plays a role in adventitious rooting (Díaz-Sala et al., 1997), and microtubules and microtubule-associated proteins are involved in adventitious root formation in several species (Abu-Abied et al., 2014, 2015a,b; Zhang et al., 2021). Differentially expressed genes in hypocotyls and epicotyls at the time of excision and during adventitious root formation are functionally associated with microtubule-associated proteins, indicating roles for microtubule dynamics in growth and the maturation-related decline of adventitious root formation in pine. Most genes show high mRNA levels in cH21, indicating a high cytoskeleton dynamic in an organ undergoing active growth and cell elongation at the time of excision. However, mRNA levels were lower for most genes in ncH91 and ncE91 (Supplementary Figure 5D). Similar to small GTP-binding proteins, the differential expression levels of vesicle trafficking and cytoskeleton dynamic genes may indicate that the expression is associated with common processes in ncE91 and ncH91, with the latter showing a high rate of cell differentiation at the time of excision. These genes were expressed following an auxin-, age-, or developmental-dependent manner during adventitious root formation in response to wounding and auxin (Figure 3D and Supplementary Figures 6D, 7D). Although the initial response to IBA was closely related in the three cutting types, the distance of the response at 6 days to auxin indicates that the differential dynamics of microtubules may be related to the maturation-related decline in adventitious root formation after the induction of rapid cell division, resulting in meristem organization (Figures 3D, 4D). Similar results have been described by Abu-Abied et al. (2014) in *Eucalyptus grandis*. A fine-tuned crosstalk between microtubules, cell walls, and auxin transport is also required for proper adventitious root induction in *Arabidopsis* (Abu-Abied et al., 2015a,b). This crosstalk could be directly or indirectly relevant to adventitious root formation in rooting-competent cuttings in response to wounding and auxin. Members of this family are involved in several organogenesis processes in response to developmental and environmental signals, including lateral root formation and root development (Lucas and Shaw, 2012; Petrovská et al., 2012; Gavrin et al., 2020). The low mRNA levels of key components involved in the stabilization of microtubules and microfilaments, such as *SCAR3*, *AIR9*, and *SPIRAL2*, or the high mRNA levels of proteins involved in the organization and orientation of the cytoskeleton, such as *AURORA* kinases, *KINESIN* and the microtubule-associated protein MAP65, in cH21 after 6 days may be related to the flexibility of the cytoskeleton needed to organize an adventitious root meristem. The different expression patterns of different family members in rooting-competent and non-competent cuttings during adventitious root formation may reflect the specificity of the responses in the various tissues and/or developmental stages.

### Expression of Genes Encoding Polar Auxin Transport Proteins Is Associated With the Presence of Exogenous Auxin in Rooting-Competent and Non-competent Stem Cuttings During Adventitious Root Formation

The requirement for polar auxin transport in the induction of adventitious root formation in pine, and the asymmetric distribution of auxin detected in rooting-competent tissues of pine after excision and during the early stages of adventitious root formation (Díaz-Sala et al., 1996; Abarca et al., 2014) indicate the involvement of proteins regulating polar auxin transport in adventitious root formation. PIN and AUX1-like auxin influx carriers are two types of proteins involved in polar auxin transport that are associated with the formation of adventitious roots in many species (da Costa et al., 2020; Li et al., 2020; Velada et al., 2020). Variations in their mRNA levels in hypocotyls and in epicotyls indicate variations in polar auxin transport, and perhaps, the redundancy of different proteins during development (Supplementary Figure 5E). These genes were mainly expressed following an association with exogenous auxin during adventitious root formation (Figure 4E). The downregulation of *AUX1* and *PIN1*, and the upregulation of *PIN2* and *PIN3*, in the presence of IBA, suggests that auxin regulates its own transport in hypocotyls and epicotyls and, as a consequence, the auxin distribution and auxin maxima are regulated by the expression levels of these genes and proteins during adventitious root formation. However, the close responses between rooting-competent and non-competent cuttings to auxin suggests that additional signaling pathways, perhaps involved in the distribution and polarity of polar auxin transport proteins in the membrane, are also involved in the variation in the auxin distribution and the auxin maxima in the rooting-competent cells compared with non-competent cells during adventitious root formation in pine (Abarca et al., 2014).

Variations in the expression levels of specific genes encoding the cell wall components involved in cell wall modifications and the maintenance of cell wall integrity, as well as cytoskeleton-related proteins were detected in rooting-competent and non-competent cuttings in response to wounding and auxin, and, perhaps, some of them were related to adventitious root induction in competent cuttings. However, the major correlation with competence for adventitious root formation was detected in a family of genes encoding proteins involved in the sensing of cell wall and membrane disturbances, such as specific RLKs and MSCs, and downstream regulators, such as members of the GTP-binding proteins. In rooting-competent tissues, the downregulation of specific RLKs or MSCs may result in a release of the signaling anchorage that maintains cell polarity and this makes rooting cells from competent hypocotyl cuttings more accommodating to modifications in the mechanical signaling from the cell wall and the reorganization of cytoskeleton in response to wounding and auxin that may shift auxin carrier polarity and auxin distribution. However, several considerations should be taken into account. Although a GRAS gene expression analysis included in the Codeset demonstrated the accuracy of the NanoString technology in this experimental system, the possibility that a probe recognizes other genes from *P. radiata* cannot be ruled out. This would explain specific variations in gene expression, especially of genes encoding cell wall and cytoskeleton proteins. This experimental system based on the rooting responses of hypocotyl and epicotyl cuttings of young seedlings simplifies studies on age- and maturation-associated loss of rooting potential. It has been used for many years and its rooting responses are well characterized (Díaz-Sala et al., 1996; Hutchison et al., 1999; Greenwood et al., 2001; Sánchez et al., 2007; Solé et al., 2008; Abarca et al., 2014; Pizarro and Díaz-Sala, 2020). However, the expression analysis of these genes using clones or genotypes of mature trees showing contrasting responses in rooting capability may shed more light on the involvement of the cell wall–plasma membrane–cytoskeleton continuum in rooting competence. Additionally, the effects of other hormones and conditions that may enhance or favor the rooting of cuttings on gene expression should also be explored. Identifying upstream regulators and downstream targets of different members of this system will help elucidate how this apparently interconnected and relatively complex regulatory network involving auxin, cell wall, RLKs, MSCs, RAC/ROPs and cytoskeleton regulates the competence of rooting cells to organize an adventitious root meristem.

## Conclusion

The expression patterns of genes encoding proteins involved in the cell wall–plasma membrane–cytoskeleton continuum in rooting-competent and non-competent cuttings in response to IBA and wounding suggest roles for the regulatory systems involving sensors of cell wall and membrane disturbances and downstream regulators belonging to the small GTP-binding family in the maturation-related decline of adventitious root formation. Their functions may be associated with specific modifications of cell wall and cytoskeleton gene expression dynamics. The molecular dissection of the mechanisms underlying the competence for adventitious root formation, along with cutting-edge technologies for analyzing multigene expression profiles, will allow the identification of an expressional signature that characterizes specific levels of regulation and factors involved in adventitious root formation. This expressional signature could be used to predict competence and may provide additional tools for competence modification. A more precise characterization of tissues used in operational programs will allow individualized management after diagnosis.

## Data Availability Statement

The original contributions presented in the study are included in the article/Supplementary Material, further inquiries can be directed to the corresponding author/s.

## Author Contributions

AP performed the plant and sample preparations, RNA extraction and NanoString analysis preparation, and contributed to the manuscript. CD-S conceptualized the work, analyzed the results, and wrote the manuscript. Both authors contributed to the article and approved the submitted version.

## Conflict of Interest

The authors declare that the research was conducted in the absence of any commercial or financial relationships that could be construed as a potential conflict of interest.

## Publisher’s Note

All claims expressed in this article are solely those of the authors and do not necessarily represent those of their affiliated organizations, or those of the publisher, the editors and the reviewers. Any product that may be evaluated in this article, or claim that may be made by its manufacturer, is not guaranteed or endorsed by the publisher.

## Footnotes

1
http://www.nanostring.com

## Supplementary Material

Supplementary Figure 1 **(A)** Expression profile of genes involved in the cell wall–plasma membrane–cytoskeleton continuum in rooting-competent hypocotyl cuttings from 21-day-old seedlings (H21) and both non-competent hypocotyl (H91) and epicotyl (E91) cuttings from 91-day-old-seedlings. RNA was extracted from the bases of hypocotyl (H) and epicotyl (E) cuttings at the time of excision (t0). An agglomerative cluster analysis was performed based on Pearson’s distances between gene expression levels and plotted as a heat map with a dendrogram of sample data. **(B)** Principal component analysis showing the gene expression data in rooting-competent hypocotyl cuttings from 21-day-old seedlings (H21) and both non-competent hypocotyl (H91) and epicotyl (E91) cuttings from 91-day-old-seedlings at the time of excision (t0).

Supplementary Figure 2 Principal component analysis showing the gene expression data in rooting-competent hypocotyl cuttings from 21-day-old seedlings (H21) and both non-competent hypocotyl (H91) and epicotyl (E91) cuttings from 91-day-old-seedlings during adventitious root formation, in cuttings treated with 10 μM indole-3-butyric-acid vs. control (mock) and during the time course at the indicated times (days) vs. t0 (time of excision).

Supplementary Figure 3 Volcano plots of a linear regression of the differential gene expression in epicotyl **(A)** and hypocotyl **(B)** cuttings from 91-day-old seedlings (E91 and H91, respectively) using the gene expression in hypocotyl cuttings from 21-day-old seedlings (H21) at the time of excision as the baseline (t0). Point colors and horizontal lines indicate various false discovery rate (FDR) thresholds.

Supplementary Figure 4 Volcano plots of a linear regression of the differential gene expression after an auxin treatment **(A)** during adventitious root formation using the gene expression of the control as the baseline, and for 1 **(B)**, 2 **(C)**, and 6 **(D)** days after treatments using gene expression at the time of excision (t0) as the baseline. Point colors and horizontal lines indicate various false discovery rate (FDR) thresholds.

Supplementary Figure 5 Expression profile of genes encoding cell wall modification **(A)**, receptor-like kinases **(B)** small GTPase-related **(C)**, cytoskeleton-related **(D)**, and auxin carrier **(E)** proteins in rooting-competent hypocotyl cuttings from 21-day-old seedlings (H21) and both non-competent hypocotyl (H91) and epicotyl (E91) cuttings from 91-day-old-seedlings. RNA was extracted from the bases of hypocotyl (H) and epicotyl (E) cuttings at the time of excision (t0). An agglomerative cluster analysis was performed based on Pearson’s distances between gene expression levels and plotted as a heat map with a dendrogram of sample and gene data.

Supplementary Figure 6 Principal component analysis-biplot showing the expression distribution of genes encoding cell wall modification **(A)**, receptor-like kinases **(B)**, small GTPase-related **(C)**, and cytoskeleton-related **(D)** proteins in 10 μM indole-3-butyric-acid-treated vs. non-treated (mock) cuttings in a two-dimension surface extracted from the principal component analysis.

Supplementary Figure 7 Principal component analysis-biplot showing the expression distributions of genes encoding cell wall modification **(A)**, receptor-like kinases **(B)**, small GTPase-related **(C)**, and cytoskeleton-related **(D)** proteins in a time course at the indicated times (days) in a two-dimension surface extracted from the principal component analysis.

Supplementary Table 1 Gene annotation and sequences for the Codeset of the NanoString probes.

Supplementary Table 2 Differential gene expression (*p* ≤ 0.1) in rooting-competent hypocotyl cuttings from 21-day-old seedlings (H21) and in both non-competent hypocotyl (H91) and epicotyl (E91) cuttings from 91-day-old-seedlings at the time of excision (t0) and during adventitious root formation, in cuttings treated with 10 μM indole-3-butyric-acid vs. control (mock) and during the time course at the indicated times (days) vs. t0 (time of excision).
