# Supplementary figures and images for: Expression Levels of Genes Encoding Proteins Involved in the Cell Wall–Plasma Membrane–Cytoskeleton Continuum Are Associated With the Maturation-Related Adventitious Rooting Competence of Pine Stem Cuttings
Source: Front Plant Sci. 2022 Jan 20;12:783783. doi: 10.3389/fpls.2021.783783 (PMC8810826; doi:10.3389/fpls.2021.783783)

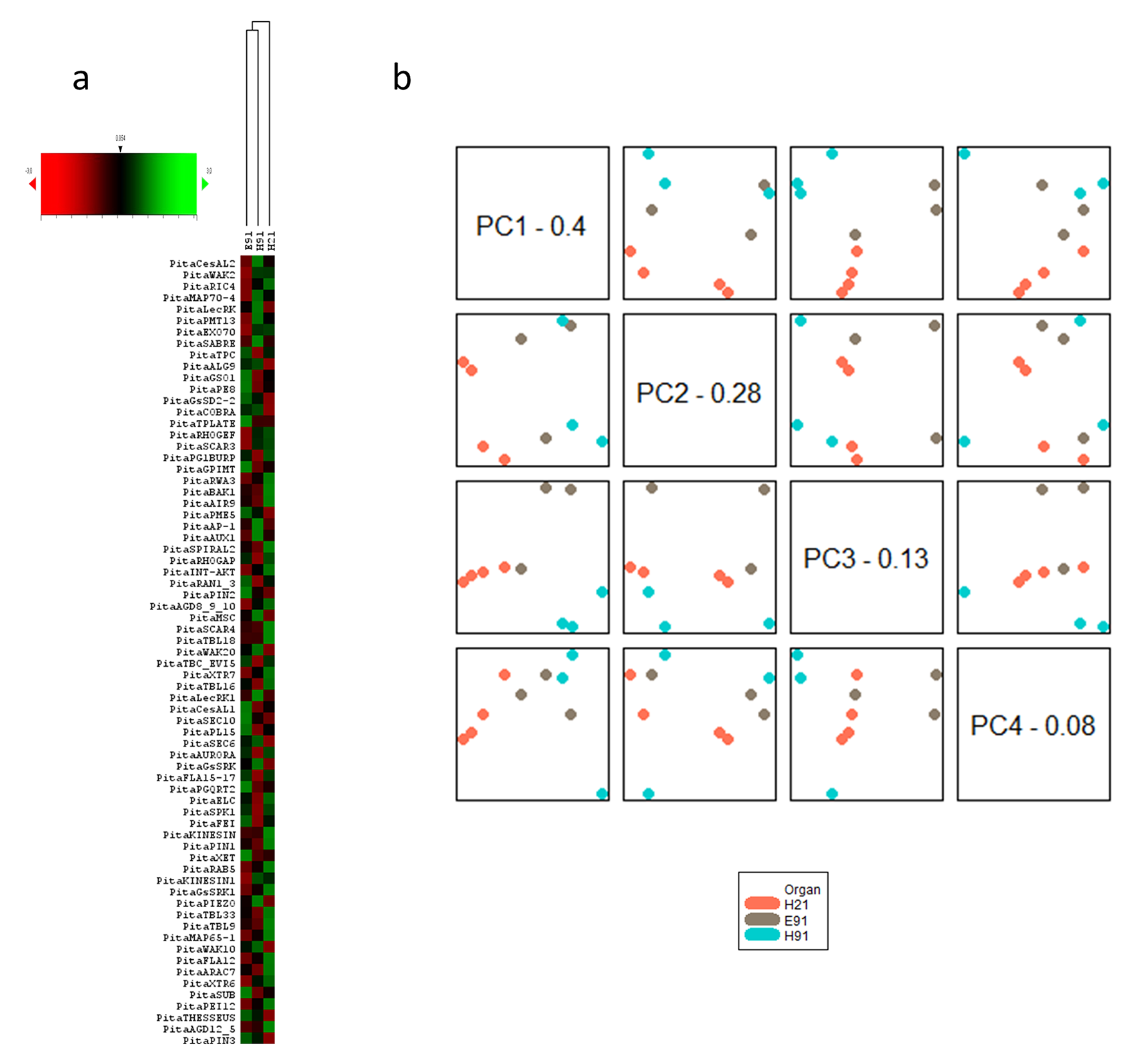

Supplement: Supplementary Figure 1 — (A) Expression profile of genes involved in the cell wall–plasma membrane–cytoskeleton continuum in rooting-competent hypocotyl cuttings from 21-day-old seedlings (H21) and both non-competent hypocotyl (H91) and epicotyl (E91) cuttings from 91-day-old-seedlings. RNA was extracted from the bases of hypocotyl (H) and epicotyl (E) cuttings at the time of excision (t0). An agglomerative cluster analysis was performed based on Pearson’s distances between gene expression levels and plotted as a heat map with a dendrogram of sample data. (B) Principal component analysis showing the gene expression data in rooting-competent hypocotyl cuttings from 21-day-old seedlings (H21) and both non-competent hypocotyl (H91) and epicotyl (E91) cuttings from 91-day-old-seedlings at the time of excision (t0). [file Image_1.TIF]

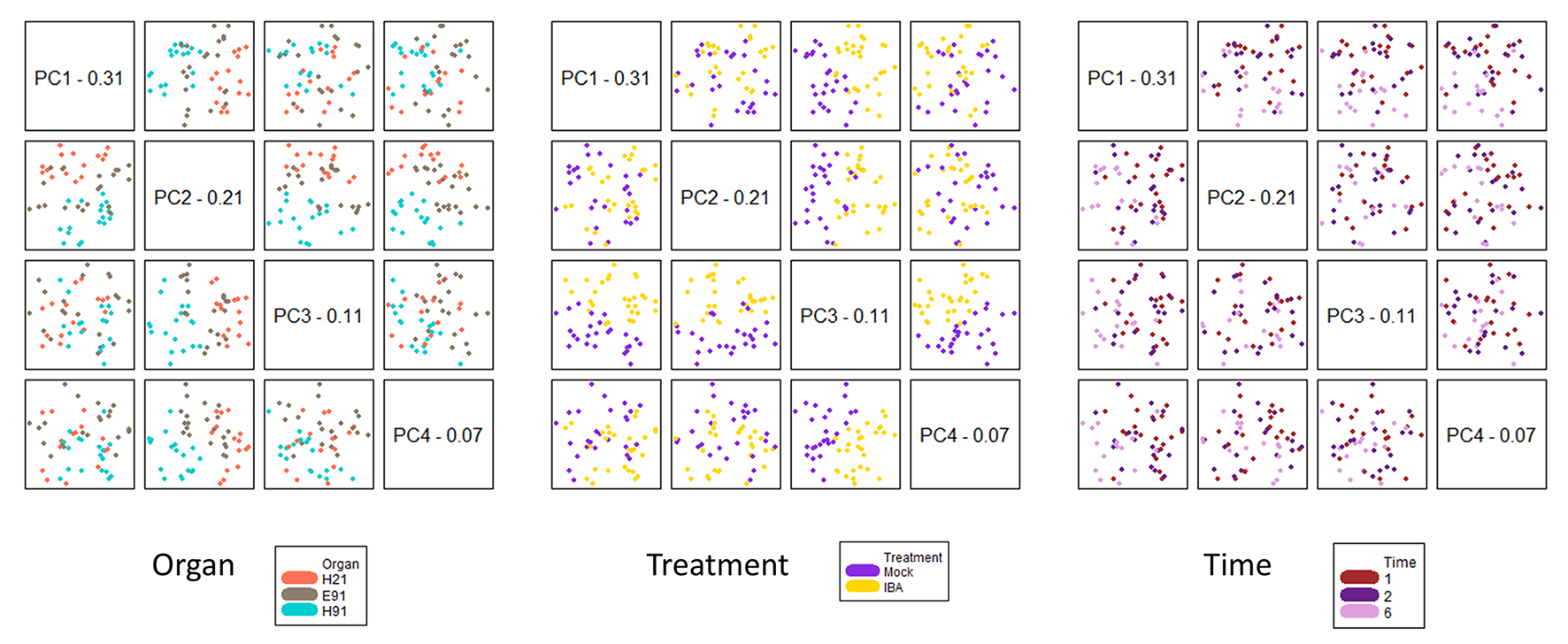

Supplement: Supplementary Figure 2 — Principal component analysis showing the gene expression data in rooting-competent hypocotyl cuttings from 21-day-old seedlings (H21) and both non-competent hypocotyl (H91) and epicotyl (E91) cuttings from 91-day-old-seedlings during adventitious root formation, in cuttings treated with 10 μM indole-3-butyric-acid vs. control (mock) and during the time course at the indicated times (days) vs. t0 (time of excision). [file Image_2.TIF]

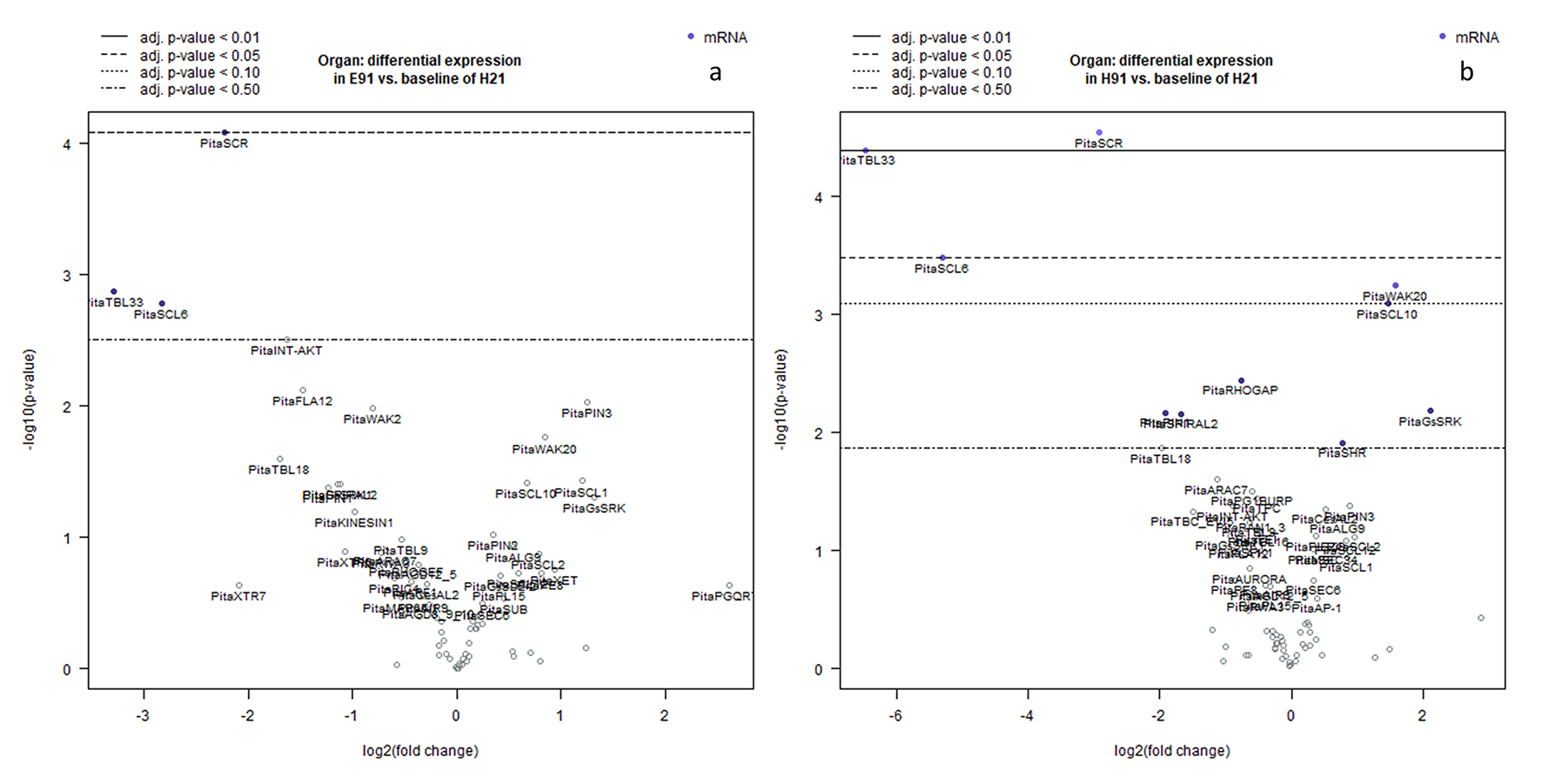

Supplement: Supplementary Figure 3 — Volcano plots of a linear regression of the differential gene expression in epicotyl (A) and hypocotyl (B) cuttings from 91-day-old seedlings (E91 and H91, respectively) using the gene expression in hypocotyl cuttings from 21-day-old seedlings (H21) at the time of excision as the baseline (t0). Point colors and horizontal lines indicate various false discovery rate (FDR) thresholds. [file Image_3.TIF]

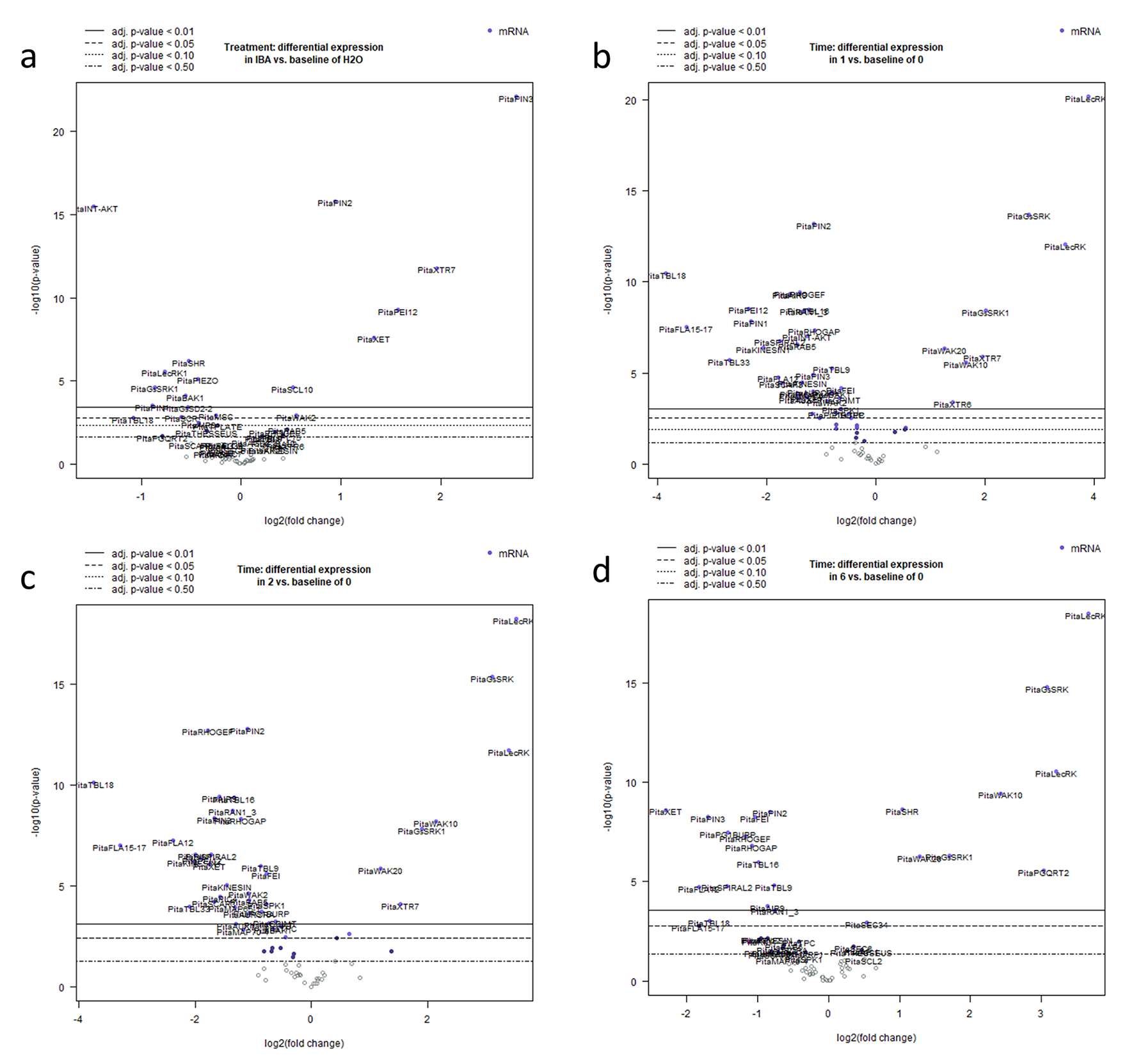

Supplement: Supplementary Figure 4 — Volcano plots of a linear regression of the differential gene expression after an auxin treatment (A) during adventitious root formation using the gene expression of the control as the baseline, and for 1 (B), 2 (C), and 6 (D) days after treatments using gene expression at the time of excision (t0) as the baseline. Point colors and horizontal lines indicate various false discovery rate (FDR) thresholds. [file Image_4.TIF]

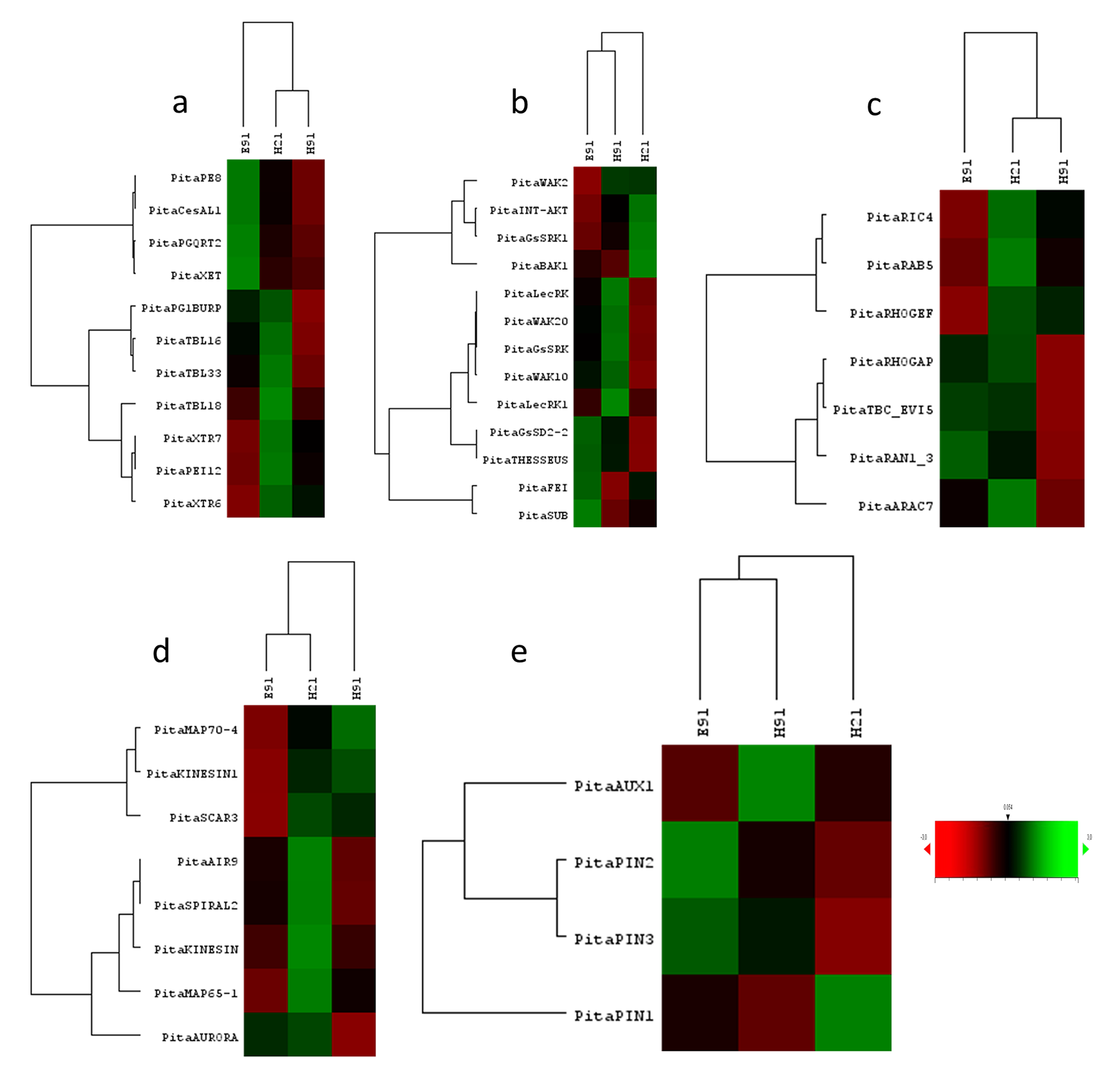

Supplement: Supplementary Figure 5 — Expression profile of genes encoding cell wall modification (A), receptor-like kinases (B) small GTPase-related (C), cytoskeleton-related (D), and auxin carrier (E) proteins in rooting-competent hypocotyl cuttings from 21-day-old seedlings (H21) and both non-competent hypocotyl (H91) and epicotyl (E91) cuttings from 91-day-old-seedlings. RNA was extracted from the bases of hypocotyl (H) and epicotyl (E) cuttings at the time of excision (t0). An agglomerative cluster analysis was performed based on Pearson’s distances between gene expression levels and plotted as a heat map with a dendrogram of sample and gene data. [file Image_5.TIF]

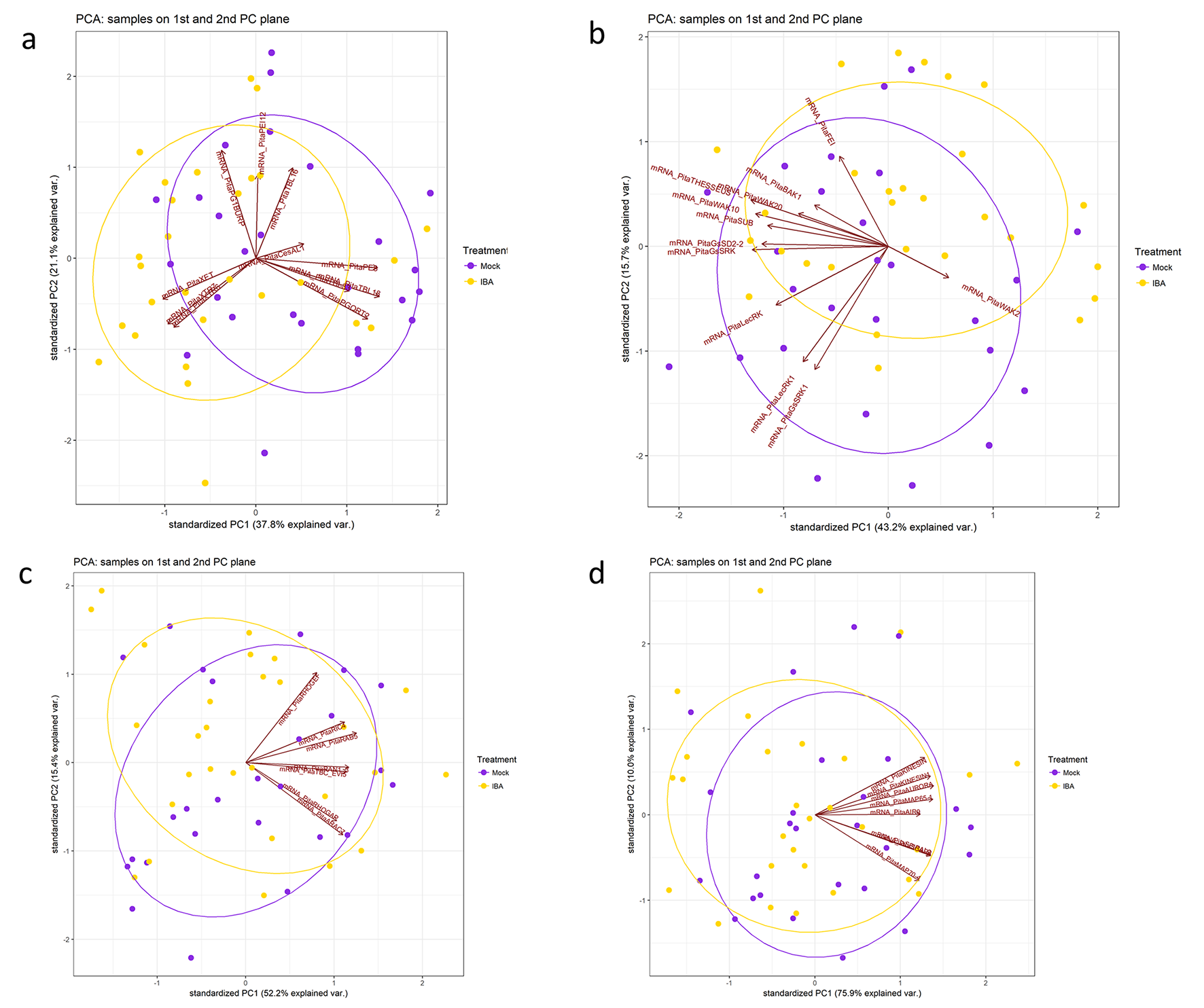

Supplement: Supplementary Figure 6 — Principal component analysis-biplot showing the expression distribution of genes encoding cell wall modification (A), receptor-like kinases (B), small GTPase-related (C), and cytoskeleton-related (D) proteins in 10 μM indole-3-butyric-acid-treated vs. non-treated (mock) cuttings in a two-dimension surface extracted from the principal component analysis. [file Image_6.TIF]

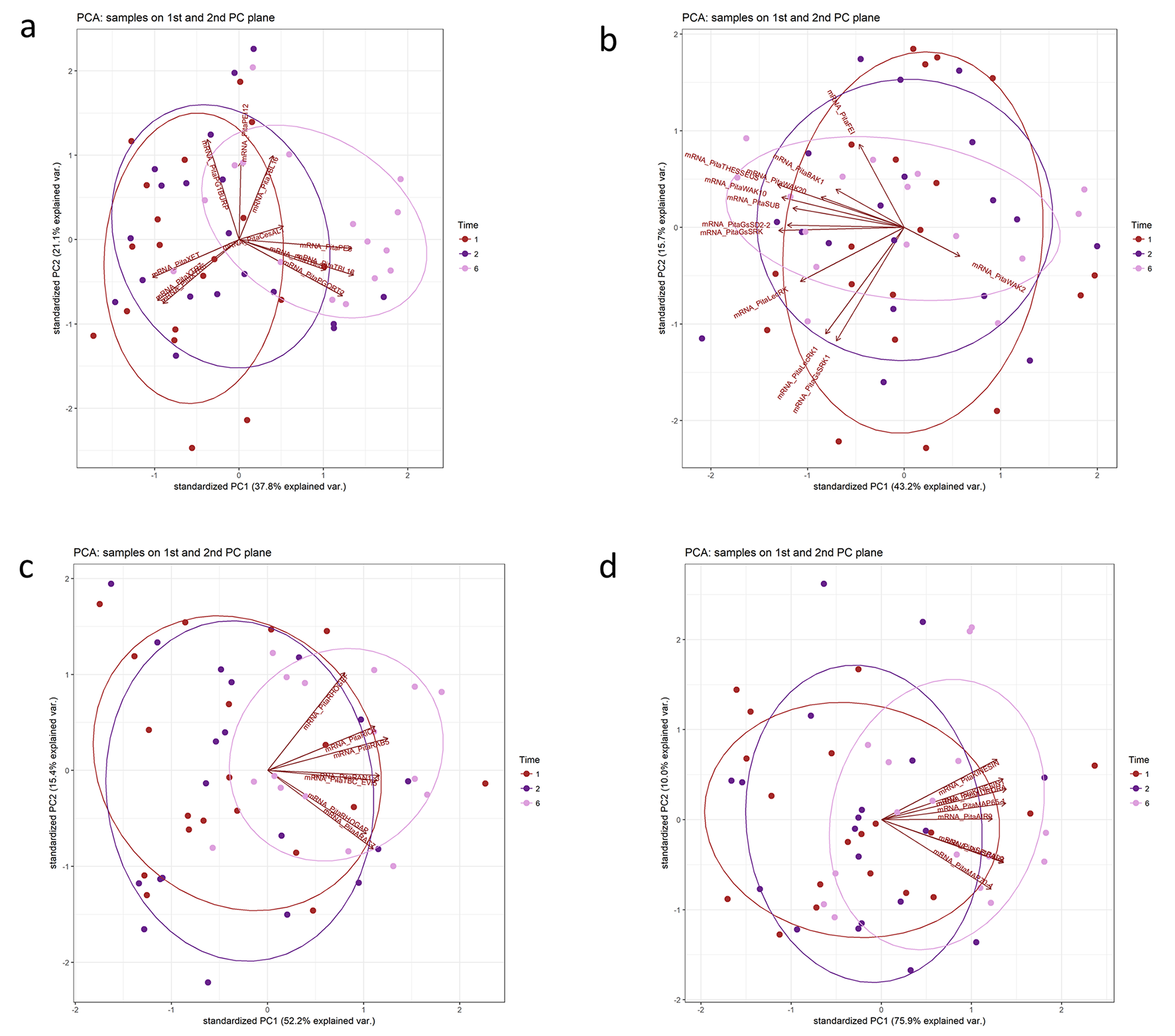

Supplement: Supplementary Figure 7 — Principal component analysis-biplot showing the expression distributions of genes encoding cell wall modification (A), receptor-like kinases (B), small GTPase-related (C), and cytoskeleton-related (D) proteins in a time course at the indicated times (days) in a two-dimension surface extracted from the principal component analysis. [file Image_7.TIF]
